# Rational Molecular Design of Complementary Self-Assembling Peptide Hydrogels

**DOI:** 10.1002/adhm.201200047

**Published:** 2012-07-12

**Authors:** Stuart Kyle, Susan H Felton, Michael J McPherson, Amalia Aggeli, Eileen Ingham

**Affiliations:** 1 Institute of Medical & Biological Engineering, Institute of Molecular & Cellular Biology, Faculty of Biological Sciences, University of LeedsLeeds, LS2 9JT, UK; 2 Centre for Molecular Nanoscience, School of Chemistry, University of LeedsLeeds, LS2 9JT, UK; 3 Astbury Centre for Structural Molecular Biology, Faculty of Biological Sciences, University of LeedsLeeds, LS2 9JT, UK

**Keywords:** complementary peptides, hydrogels, scaffolds, self-assembly

Over the past 20 years self-assembling peptides have been shown to be of value as scaffolds in the field of tissue engineering.^1^ Self-assembling peptides offer many advantages. Their complex nanostructures can be easily synthesised from simple precursors. It is possible to control their chemical functionality to make them environmentally responsive, for example to pH or temperature. Their biological activity can be modified by the inclusion of bioactive sequences, such as cell adhesion motifs. The development of distinct self-assembling peptide systems with various functionalities will be important for tailoring and optimising biological performance, especially in relation to active scaffolds for tissue engineering and regenerative medicine. A variety of peptides have been rationally designed to spontaneously self-assemble into hierarchical structures in solution and in response to specific physico-chemical environmental triggers.^2^ Under controlled conditions self-assembling peptides undergo one-dimensional self-assembly forming single molecule thick, micrometer-long beta-sheet nanotapes. Further assembly can be induced such that the nanotapes stack in pairs to form ribbons which further assemble to form fibrils and pairs of fibrils can then entwine to form fibres. Self-assembling peptide hydrogels represent valuable systems for delivering cells embedded within a scaffold. Single component self-assembling systems allow for sol-gel transitions in which the peptide is monomeric, for example within a syringe at room temperature at an injection site, but self-assembles into a gel state at 37 °C upon injection. An alternative approach is to use a binary system in which two separate complementary peptides that cannot self-assemble individually are co-delivered to the injection site and, upon mixing, self-assemble into a gel state. Such systems have been previously documented.2k The Aggeli group have designed over twenty self-assembling peptides to self-assemble under various physico-chemical conditions. In earlier work we exploited the propensity of peptide chains to self-assemble via intermolecular hydrogen bonding into β-sheet structures,2a,b,i and demonstrated that oligopeptides can be designed to self-assemble into micrometer-long β-sheet tapes.2a In order to produce β-sheet tapes in solution, we used the following working criteria: (i) cross-strand attractive forces (hydrophobic, electrostatic, hydrogen-bonding) between side-chains, (ii) lateral recognition between adjacent β-strands to constrain their self-assembly to one dimension, and avoid heterogeneous aggregated β-sheet structures, and (iii) strong adhesion of solvent to the surface of the tapes to control solubility.2a,b,i Based on these criteria, a *de* novo 11-residue peptide DN1 (later called P_11_-2; CH_3_CO-Gln-Gln-Arg-Phe-Gln-Trp-Gln-Phe-Glu-Gln-Gln-NH_2_) was designed to form β-sheet polymer tapes in water. Subsequently, the P_11_-family of β-sheet-forming self-assembling peptides were rationally designed. Subtle changes in peptide design can lead to control of mechanical properties, surface chemistry, morphology, bioactivity and responsiveness of the self-assembly of these materials to external chemical triggers (pH, ionic strength).2c,d

In this Communication, we report on a binary peptide system P_11_-13 and P_11_-14 including biophysical analysis and cell-based studies, and we have compared cell behaviour to that shown by a previously reported, single component self-assembling peptide P_11_-4 (CH_3_CO-Gln-Gln-Arg-Phe-Glu-Trp-Glu-Phe-Glu-Gln-Gln-NH_2_).2c,d The complementary peptides P_11_-13 (CH_3_CO-Glu-Gln-Glu-Phe-Glu-Trp-Glu-Phe-Glu-Gln-Glu-NH_2_) and P_11_-14 (CH_3_CO-Gln-Gln-Orn-Phe-Orn-Trp-Orn-Phe-Orn-Gln-Gln-NH_2_, where Orn represents ornithine) are not based on any native protein having been rationally designed. The choice of residues allows for various intermolecular arrangements which lead to favourable contacts between each peptide strand. The hydrophobic residues, Phe and Trp, provide hydrophobic interactions between side-chains and intermolecular recognition by π−π interactions, Orn and Glu provide strong coulombic and complementary electrostatic interactions, and Gln, Orn and Glu side-chains make one surface of the β-sheet more hydrophilic than the other. We highlight the importance of rational molecular design of self-assembling peptide-based materials. One area of interest is the development of scaffolds for tissue engineering, and we have examined this through studies of the capacity of the peptide hydrogel to support cell proliferation. Alternative proposed applications for the binary system include a scaffold for controlled drug delivery, as an injectable therapeutic, as antimicrobial gels or templates for mineralization.

The complementary peptides, P_11_-13 and P_11_-14, were designed to each be monomeric and in the fluid state under physiological conditions (140 mM NaCl, pH 7.4) at peptide concentrations greater than 10 mg mL^−1^. P_11_-13 and P_11_-14 have negative and positive charges respectively under these conditions. Upon mixing of equal quantities a self-supporting hydrogel instantaneously formed in cell culture medium (**Figure** **1**a) (See also Supporting Information **Movie S1**) due to the complementary ionic bonding between the positively and negatively charged residues of the peptides. Moreover, the assembly of these peptides was not pH reversible.

**Figure 1:**
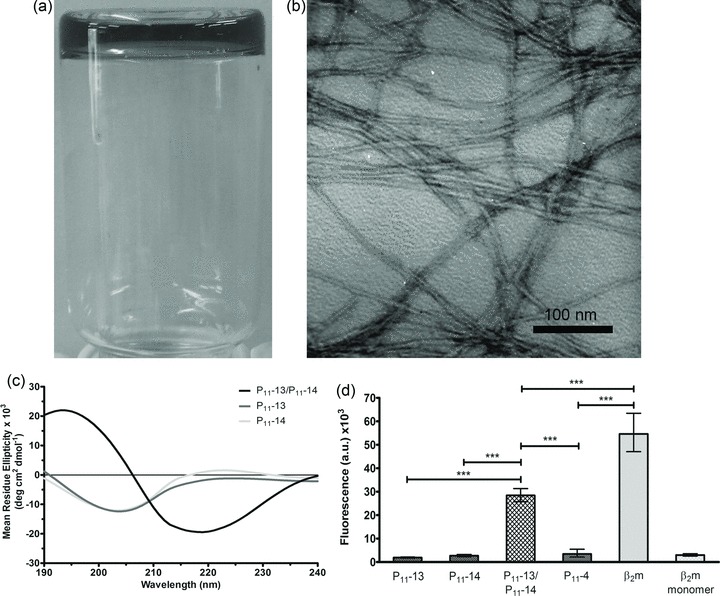
a) Self-assembly of complementary peptides P_11_-13 and P_11_-14 which formed self-supporting transparent hydrogels in physiological conditions at pH 7.4 in 140 mM NaCl at peptide concentrations of 6.3 mM in cell culture medium. b) TEM of P_11_-13/P_11_-14 peptide fibrils and fibres, prepared at a concentration of 6.3 mM in 140 mM NaCl and diluted to 100 μM with distilled water (pH 7.4). Fibres are relatively straight and long with fibre-like junctions and networks clearly seen. c) Far UV CD spectra of P_11_-13, P_11_-14 and P_11_-13/P_11_-14 at pH 7.4. Peptides were prepared at 6.3 mM in 140 mM NaCl and diluted to 100 μM with distilled water. A characteristic negative minimum at around 220 nm and positive maximum at around 198 nm was indicative of a β-sheet conformation for P_11_-13/P_11_-14. A negative minimum at around 200 nm and positive maximum at around 222 nm was indicative of random coil configuration for P_11_-13 and P_11_-14, confirming their unstructured monomeric nature. d) Thioflavin T fluorescence of P_11_-13 and P_11_-14 monomers, P_11_-13/P_11_-14 hydrogel and P_11_-4 hydrogel (6.3 mM, 140 mM NaCl, pH 7.4) compared to a known amyloidogenic agent, β_2_ microglobulin (β_2_m). *** represents a significant difference as determined by one-way ANOVA (*p* < 0.001).

The nanostructures formed upon self-assembly of P_11_-13 and P_11_-14 were examined by transmission electron microscopy (TEM). This revealed long P_11_-13/P_11_-14 peptide fibrils, some being greater than 2 μm in length. Fibrils were noticeably bundled in relatively straight lines and fibrillar networks were also apparent. Fibril widths ranged from 10 to 20 nm in diameter (Figure 1b).

Circular dichroism (CD) was used to investigate the secondary structure characteristics of P_11_-13/P_11_-14 within the hydrogel, and of P_11_-13 and P_11_-14 monomeric forms. A characteristic β-sheet conformation was adopted for P_11_-13/P_11_-14 as shown by a negative minimum at around 220 nm and a positive maximum at around 198 nm (Figure 1c). P_11_-13 and P_11_-14 monomers by contrast showed a characteristic random coil conformation with a positive maximum at around 222 nm and a negative minimum at around 200 nm.

It is known that amyloid fibrils formed by certain proteins can be toxic, yet fibrils formed by some synthetic peptides are non toxic showing no effect on cell viability. Hence careful molecular design of synthetic peptide sequences is important, for example in avoiding amino acid sequences known to promote toxic amyloid fibrils under certain conditions. The thioflavin T assay was used to determine whether the P_11_-13 and P_11_-14 monomers, and P_11_-13/P_11_-14 hydrogel displayed any amyloidogenic character (Figure 1d). P_11_-13 and P_11_-14 monomers were found to be substantially less amyloidogenic when compared to a known amyloidogenic protein, β_2_-microglobulin. The P_11_-13/P_11_-14 hydrogel was significantly more amyloidogenic than the individual P_11_-13 and P_11_-14 monomers (*p* < 0.001). The peptide hydrogel was, however, significantly less amyloidogenic than β_2_-microglobulin (*p* < 0.001), a well characterised amyloidogenic protein. Similarly for P_11_-4, thioflavin T fluorescence was found to be significantly lower than that of β_2_-microglobulin (*p* < 0.001). P_11_-4 and β_2_m monomer (no fibril aggregation) showed similar levels of thioflavin T fluorescence. Of course the results obtained from this preliminary *in vitro* thioflavin T assay may not translate to an *in vivo* model, and work is currently underway *in vivo* in a mouse model.

Biological testing was performed on P_11_-13/P_11_-14 in comparison to P_11_-4 hydrogel and collagen type I. Contact cytotoxicity assays were used to assess biocompatibility with primary human dermal fibroblasts. Microscopic analysis revealed that primary human dermal fibroblasts grew up to and in contact with P_11_-13/P_11_-14 (Figure 2a) and P_11_-4 (Figure 2b) and there was no evidence of cytotoxicity. Fibroblasts could clearly be seen attaching to and aligning along the periphery of the hydrogel, with some infiltrating onto the hydrogel. This was comparable behaviour to that observed for type I collagen (negative control), which is not cytotoxic (Figure 2c). By contrast, cyanoacrylate (positive control) caused cell lysis and agglomerated cell remnants were observed (Figure 2d).

**Figure 2:**
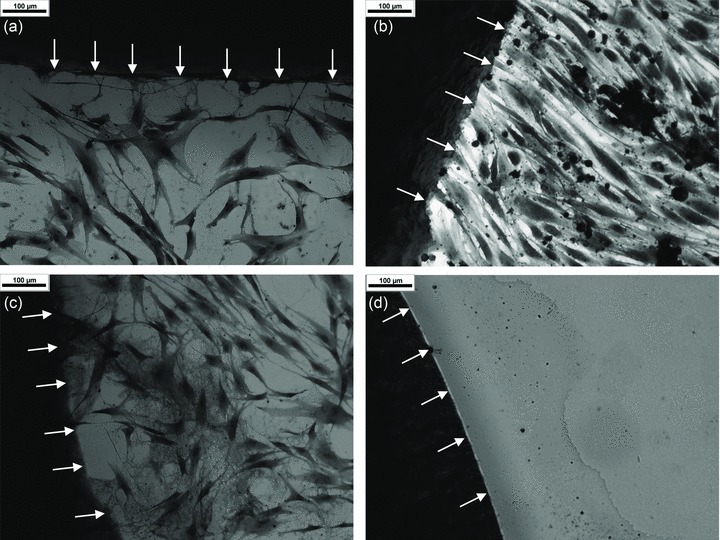
Contact cytotoxicity assay of: a) P_11_-13/P_11_-14 and b) P_11_-4 hydrogels at 30 mg mL^−1^ in DMEM plus supplements (pH 7.4) following 48 h incubation with primary human dermal fibroblasts. c) Collagen type I (negative control). d) Cyanoacrylate (positive control). White arrows indicate the periphery of the material that the cells grew in contact with.

The next step was to determine whether the P_11_-13/P_11_-14 hydrogel could support 3D cell proliferation of human dermal fibroblasts over 28 days. Primary human dermal fibroblasts were seeded at a cell density of 5 × 10^4^ cells mL^−1^ within P_11_-13/P_11_-14, P_11_-4 and collagen hydrogels. The number of living cells were determined over a 28 day period by using the ATPLite-M assay. Different concentrations of P_11_-13/P_11_-14 were tested from 5 mg mL^−1^ to 30 mg mL^−1^, and P_11_-4 was used at 30 mg mL^−1^. The number of cells decreased over the 28 day culture period at all P_11_-13/P_11_-14 peptide concentrations (Figure 3). By comparison, the number of cells increased over the 28 days when grown in P_11_-4 and collagen matrices. There was no significant difference in cellular ATP levels between the different P_11_-13/P_11_-14 peptide concentrations from day 0 to day 21. However, at day 28 there were significantly higher numbers of cells in the 5 mg mL^−1^ peptide hydrogels compared to the 30 mg mL^−1^ hydrogels. It was evident that in P_11_-13/P_11_-14 hydrogels the number of viable cells declined over the 28 period but that the decline was less pronounced in the lower peptide concentration samples. A significant difference was observed between all P_11_-13/P_11_-14 peptide concentrations and the P_11_-4 and collagen from 14 days onwards (*p* < 0.001).

**Figure 3:**
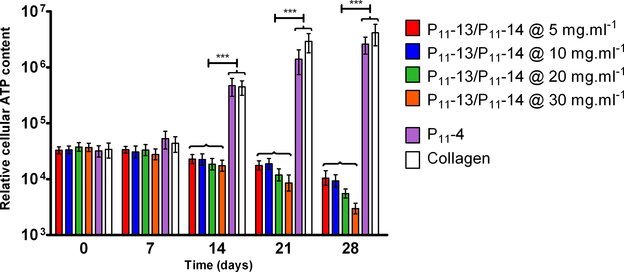
Proliferation of primary human dermal fibroblasts within P_11_-13/P_11_-14 hydrogel at peptide concentrations ranging from 5 mg mL^−1^ to 30 mg mL^−1^, P_11_-4 at a peptide concentration of 30 mg mL^−1^, and collagen type I over 28 days. Data are expressed as mean values (n = 3) ± 95% confidence limits. *** represents significant increases as determined by ANOVA.

Sections of P_11_-13/P_11_-14 and P_11_-4 hydrogels were stained with haematoxylin and eosin, with and without the encapsulation of primary human dermal fibroblasts. Histological analysis revealed very different structures. To our knowledge similar structures have not been reported for other self-assembling peptide systems. Histologically, P_11_-13/P_11_-14 hydrogel showed an ordered array of large fibre-like networks with smaller interconnecting fibrils. Images were taken at different magnifications (Figure 4a, b) and revealed a repetitive brick-like architecture that seemed to provide little opportunity for cell migration. In the presence of cells a similar brick-like structures was also apparent, however, it was observed that no cell nuclei were visible but rather small dense areas of black material were seen. These were only visible at the periphery of the hydrogel, as indicated by the black arrows (Figure 4c, d). These structures were not seen in any of the samples of P_11_-13/P_11_-14 without cells (Figure 4a, b), suggesting that these black structures were condensed dead cells. A very different histoarchitecture was seen with P_11_-4 compared with the complementary peptide system (Figure 4e, f). Without cells, an ordered array of fibrillar entanglements was observed. In the presence of cells, a less ordered fibrillar network was seen. It was clear that cell nuclei (red arrows) and neo-extracellular matrix deposition within P_11_-4 occurred at day 14 and the presence of P_11_-4 matrix degradation was also noted (Figure 4f) when compared with a control P_11_-4 matrix at day 14 (Figure 4e). This indicated that primary human dermal fibroblasts used the P_11_-4 matrix as a temporary scaffold over the 14 days to deposit ECM matrix. This highlights the range of structures obtained and significant differences on cell behaviour from relatively modest changes in molecular design.

**Figure 4:**
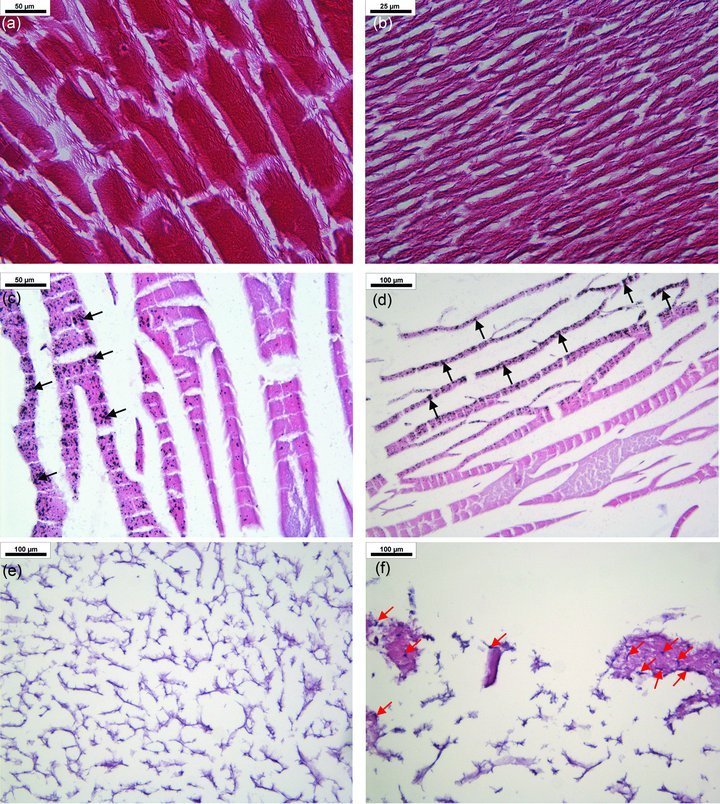
Histological images of: a–d) P_11_-13/P_11_-14 and e–f) P_11_-4 hydrogels stained with haematoxylin and eosin (H&E). a–b) No cells were seeded within the matrix. Larger fibres can be seen surrounded by smaller fibres/fibrils. Regions of the matrix appeared extremely ordered with similar sized larger and smaller fibres/fibrils, particularly evident in (a) and (b). c–d) P_11_-13/P_11_-14 hydrogel after 14 days of culture with primary human dermal fibroblasts. Black arrows indicate possible cell remnants of black, circular aggregates on some fibres. These were most evident at the periphery of the hydrogel and were not seen in no cell samples. P_11_-4 hydrogel (e) without and (f) with primary human dermal fibroblasts (red arrows) after 14 days of culture. Neo-ECM deposition occurred at the periphery of the P_11_-4 matrix, together with peptide fibril reorganisation as observed in (f).

The unusual structural organisation in the P_11_-13/P_11_-14 hydrogel may adversely influence cells perhaps by preventing effective interactions with the matrix or interfering with cell migration or cellular remodelling of the matrix. One study found that human dermal fibroblasts in compressed collagen matrices at an initial cell seeding density of 1 × 10^6^ cells mL^−1^ showed rapid proliferation, with a doubling time of 2 days in a collagen matrix with a Young's modulus of 2240 kPa.^3^ This group used a significantly higher initial cell density than was used here (1 × 10^6^ cf. 5 × 10^4^) and this may affect the ability of the cells to make interactions with other cells perhaps due to either physicochemical or structural features of the matrix that prevent effective cell migration thus leading to increased apoptosis and a decline in cell numbers over time. The fact that such effects were more pronounced at higher peptide concentrations would support this proposal. The *in vivo* environment is dynamic with a variety of forces generated on cells and tissue, and it may therefore be necessary to provide dynamic forces such as compression and tension into the P_11_-13/P_11_-14 peptide system.

In summary, complementary self-assembling peptides comprising the unimers P_11_-13 and P_11_-14, have been designed. Upon equimolar mixing these instantaneously form a translucent self-supporting hydrogel. We compared this system with a previously reported single peptide self-assembling system, P_11_-4. Nanofibre entanglements were observed by TEM while CD showed, as expected, that P_11_-13/P_11_-14 hydrogel adopted a β-sheet conformation while the unimer solutions adopted a random coil conformation. Preliminary *in vitro* amyloidogenicity studies, indicated that P_11_-13/P_11_-14 hydrogels were more amyloidogenic than P_11_-4, but statistically less amyloidogenic than a known amyloidogenic protein, β_2_-microglobulin. Both P_11_-13/P_11_-14 and P_11_-4 hydrogels were cytocompatible with primary human dermal fibroblasts. However, while these cells remained viable and proliferated over 28 days in a P_11_-4 3D hydrogel, with P_11_-13/P_11_-14 cell numbers declined over 28 days. Histological differences were also noted between the two peptide systems. P_11_-13/P_11_-14 hydrogels showed a brick-like network of large fibres surrounded by smaller interconnecting fibres where cell remnants were visible, whereas P_11_-4 showed a much less dense network of fibres and the cells elaborated neomatrix over 14 days. The results suggest that P_11_-4 is a model scaffold for the 3D culture of primary human dermal fibroblasts. The properties of P_11_-13/P_11_-14 result in hydrogels that are not cytotoxic, but which do not support proliferation of this cell type and are thus likely to be better suited to other healthcare applications such as antimicrobial gels, as injectable therapeutics, as templates for mineralization, or for drug delivery. It is also likely that further design to reduce the number of charged residues and the addition of bioactive motifs may result in functionality to support cell growth. It is clear that molecular design criteria can influence the properties of peptide materials and ultimately affect biomaterials applications.

## Experimental Section

*Self-assembly studies of P_11_-13/P_11_-14*: Unless otherwise stated peptides P_11_-13 and P_11_-14 (NeoMPS Groupe SNPE, Strasbourg, France) were prepared at concentrations of 10 mg mL^−1^ in 140 mM NaCl, pH 7.4 with pH adjustment using 1 M NaOH. To visually observe the self-assembly equal volumes of the peptides were mixed in DMEM.

*Microscopy*: Aliquots (100 μL) of P_11_-13 and P_11_-14 were mixed and agitated for 15 seconds, sonicated in an ultrasonic water bath for 15 minutes at a frequency of 45 kHz and left at 25 °C for 24 hours. The peptide hydrogels were then diluted to 100 μM in water immediately before application to a glow-discharged, carbon coated, 400 hexagonal mesh copper grid. Four separate samples of hydrogel were used for TEM analysis and each sample was performed in triplicate.

*CD UV spectroscopy*: Equal quantities (100 μL) of P_11_-13 and P_11_-14 were mixed, sonicated and left to equilibrate as for TEM analysis. P_11_-13 and P_11_-14 monomers, and P_11_-13/P_11_-14 hydrogel were diluted to a concentration of 100 μM with distilled water and equilibrated at 25 °C for 24 hours prior to analysis. Samples were analysed in quartz cuvettes (Hellma®) with a path length of 1 mm. Mean residual ellipticity readings were taken in the far UV region of the spectrum, (190-240 nm) using a Jasco J-750 spectropolarimeter. Each spectrum was the average of 5 scans with a step resolution of 0.5 nm, scan speed 50 nm min^−1^, response time of 1 second and a sensitivity of 50 m° at 20 °C. Blank readings taken for each sample was subtracted from the data from the peptide samples.

*Thioflavin T assay*: P_11_-13/P_11_-14 and P_11_-4 were prepared at a concentration of 10 mg mL^−1^ in 140 mM NaCl in deionised water at pH 7.4. The pH of P_11_-13 and P_11_-4 were adjusted to 7.4 using 1 M NaOH (2 μL). P_11_-13 (1 μL) was placed into a flat-bottomed 96-well plate to which P_11_-14 (1 μL) was added and both solutions were mixed immediately. To other wells, P_11_-13 and P_11_-14 monomers (2 μL), and P_11_-4 hydrogel (2 μL) were added. Controls used were β_2_-microglobulin aggregates and β_2_-microglobulin monomers. A blank sample of water alone was also analysed and subtracted from each peptide and control sample. Each sample (2 μL) was diluted into Thioflavin T buffer (200 μL; 10 μM ThT in 0.5 M Tris-HCl, pH 8.0). The fluorescence was monitored using the FLUOstar OPTIMA (BMG Labtechnologies Ltd, UK) with excitation at 444 nm and emission at 480 nm. Samples were measured in triplicate.

*Cell culture*: Primary human dermal fibroblasts were purchased from Cascade Biologics (Nottingham, United Kingdom). Cells were cultured using Dulbecco's-modified Eagle's medium (DMEM) supplemented with 10% (v/v) fetal calf serum, 1 mM L-glutamine, 100 U mL^−1^ penicillin and 100 μg mL^−1^ streptomycin at 37 °C in 5% (v/v) CO_2_ in air, with medium changes every two days.

*Preparation of cellular collagen type I gels*: Extracted and purified rat tail collagen (3 mL; 5.2 mg mL^−1^) was transferred to a sterile tube to which 5 mL 2 × DMEM containing supplements (20% (v/v) fetal calf serum, 20 μM L-glutamine, 200 U mL^−1^ penicillin and 200 μg mL^−1^ streptomycin) was added with gentle agitation. Sterile sodium hydroxide (1 mL; 0.1 M) was then added and agitation was continued. Cell suspensions in DMEM (1 mL) were prepared at 5 × 10^5^ cells mL^−1^ which is 10× the final cell density for cell proliferation studies after the addition of collagen/NaOH/medium. Samples were aliquoted into 6-well plates (500 μL) or 96-well plates (100 μL), and incubated for 20 minutes at 37 °C in 5% (v/v) in CO_2_ in air to allow collagen gels to polymerise.

*Contact cytotoxicity assay*: P_11_-13/P_11_-14 and P_11_-4 hydrogels were prepared at 30 mg mL^−1^ in 1× DMEM plus supplements. Collagen type I (∼250 μL) was added to the centre of wells in a 6-well plate, allowed to polymerise for 20 minutes and P_11_-4 (500 μL) was then added over the collagen type I which acted as an attachment site. Collagen type I was not needed as an attachment aid for the complementary peptides due to the robust, nature of the hydrogel and so P_11_-13 (100 μL) and P_11_-14 (100 μL) were mixed and applied directly to the centre of wells in 6-well plates. Collagen type I (500 μL) was added to wells of another 6-well plate as a cytotoxic negative control. Cyanoacrylate (∼500 μL) was added to wells of another 6-well plate as a cytotoxic positive control. Primary human fibroblasts were seeded at a density of 5 × 10^5^ cells per well in 2 mL 1× DMEM plus supplements. Plates were incubated at 37 °C in 5% (v/v) CO_2_ in air for 48 hours. All samples were tested in triplicate.

*Cell proliferation studies*: Sterile (Gamma irradiated at 25 kGy, Isotron Ltd.) P_11_-13 and P_11_-14 were prepared at peptide concentrations of 6.25, 12.5, 25 and 37.5 mg mL^−1^ in DMEM supplemented with 10% (v/v) fetal calf serum, 1% (v/v) 1 mM L-glutamine and penicillin (100 U mL^−1^) and streptomycin (100 μg mL^−1^). The pH was adjusted with 1 M NaOH until a rose pink colour reappeared due to the DMEM phenol red indicator. Peptide hydrogels were adjusted to final peptide concentrations of 5, 10, 20 and 30 mg mL^−1^ by the addition of a freshly prepared cell suspension in DMEM to P_11_-14 peptide solution. The same volume of DMEM without cells was added to P_11_-13 as a control. A sample (1 mL) of equal concentration of P_11_-13 was mixed with 1 mL P_11_-14 containing cells to give a cell density of 5 × 10^4^ cells mL^−1^ in the P_11_-13/P_11_-14 hydrogel. Sterile P_11_-4 was prepared at a concentration of 37.5 mg mL^−1^ in DMEM supplemented as above. The hydrogels were adjusted to a peptide concentration of 30 mg mL^−1^ by the addition of a freshly prepared cell suspension, with gentle agitation. Typically, an initial hydrogel volume of 1.5 mL was prepared at a peptide concentration of 37.5 mg mL^−1^ to which either DMEM (375 μL) as a control or DMEM with cells (375 μL) was added to give a final density of 5 × 10^4^ cells mL^−1^. The various hydrogel samples were transferred to a flat bottomed 96 well plate (100 μL per well). Collagen gels were also prepared as described above as controls. Collagen with and without cells was pipetted into three 96 well plates (100 μL per well). The suspensions were allowed to gel for 3 hours at 37 °C in an atmosphere of 5% (v/v) CO_2_ in air. After this time, 100 μL 1× DMEM plus supplements was added on top of the gel surface. The medium was replaced every 2 days. Three 96 well plates were prepared, containing three replicates of peptide hydrogel or collagen for each time point of 0, 7, 14, 21 and 28 days. The proliferation capacity of primary human fibroblasts within the P_11_-13/P_11_-14 and P_11_-4 matrices was assessed over 28 days. Total cell number was recorded at 7 day intervals by measuring the ATP concentration of metabolically active cells using the ATPLite-M® assay (Perkin Elmer).

*Histology*: P_11_-13 and P_11_-14 at 10 mg mL^−1^ in DMEM plus supplements were mixed to form a gel (500 μL). The pH of P_11_-13 was adjusted to pH 7.4 prior to mixing using 1 M NaOH (2 μL). P_11_-4 hydrogel was prepared at a concentration of 37.5 mg mL^−1^ in DMEM plus supplements. Hydrogels were allowed to form in 12-well cell culture inserts which acted as a support device. Primary human dermal fibroblast were seeded into the peptide matrix at a density of 5 × 10^4^ cells mL^−1^ and cultured for 14 days. P_11_-13/P_11_-14 and P_11_-4 hydrogels containing no cells were used as a control. P_11_-13/P_11_-14 hydrogels were fixed in 10% (v/v) neutral buffered formalin for 1 hour and P_11_-4 hydrogel was fixed using 2% (w/v) 1-ethyl-3-(3-dimethylaminopropyl)carbodiimide (EDC) and 1 mM *N*-hydroxysuccinimide (NHS) for 3 hours. Specimens were then dehydrated and segmented into three then embedded in paraffin wax. Serial sections, encompassing the entire peptide matrix were cut using a microtome at 9 μm, mounted onto glass slides and stained with haematoxylin and eosin to evaluate histoarchitecture. All histological sections were viewed using an Olympus BX51 upright light microscope. Images were captured using an Evolution MP colour digital camera and controlled through Image Pro Plus imaging software, version 6.1.

*Statistical analysis*: All numerical data was analysed using GraphPad Prism. Data are shown as mean values ± 95% confidence limits. Statistical significance was assessed by one-way analysis of variance (ANOVA), using the mean value of each sample group and the minimum significance difference (MSD) at *p* = 0.05 using a Tukey test. *p* values of less than 0.05 were considered significant.

Please note: In the Supporting Information Movie S1 shows real-time self-assembly of complementary self-assembling peptides P_11_-13 and P_11_-14.

## Supporting Information

Supporting Information is available from the Wiley Online Library or from the author.
